# Personalized colorectal cancer screening: study protocol of a mixed-methods study on the effectiveness of tailored intervals based on prior f-Hb concentration in a fit-based colorectal cancer screening program (PERFECT-FIT)

**DOI:** 10.1186/s12876-023-02670-1

**Published:** 2023-02-22

**Authors:** Emilie C. H. Breekveldt, Esther Toes-Zoutendijk, Lucie de Jonge, Manon C. W. Spaander, Evelien Dekker, Folkert J. van Kemenade, Anneke J. van Vuuren, Christian R. B. Ramakers, Iris D. Nagtegaal, Monique E. van Leerdam, Iris Lansdorp-Vogelaar

**Affiliations:** 1 Department of Public Health, Erasmus MC University Medical Center, Rotterdam, Dr. Molewaterplein 40, 3015GD Rotterdam, The Netherlands; 2 Department of Gastroenterology and Hepatology, Netherlands Cancer Institute – Antoni van Leeuwenhoek Hospital, Amsterdam, The Netherlands; 3 Department of Gastroenterology and Hepatology, Erasmus MC, University Medical Centre Rotterdam, Rotterdam, The Netherlands; 4 Department of Gastroenterology and Hepatology, Amsterdam University Medical Centre – Location AMC, Amsterdam, The Netherlands; 5 Department of Pathology, Erasmus MC, University Medical Centre Rotterdam, Rotterdam, The Netherlands; 6 Department of Clinical Chemistry, Erasmus MC, University Medical Centre Rotterdam, Rotterdam, The Netherlands; 7 Department of Pathology, Radboud University Medical Centre, Nijmegen, The Netherlands; 8 Department of Gastroenterology and Hepatology, Leiden University Medical Center, Leiden, the Netherlands

**Keywords:** Colorectal cancer, Colorectal neoplasia, Colonoscopy, Colorectal cancer screening, Fecal immunochemical testing

## Abstract

**Background:**

In 2014, the national population-based colorectal cancer (CRC) screening program was implemented in the Netherlands. Biennial fecal immunochemical testing (FIT) for hemoglobin (Hb) is used at a cut-off of 47 µg Hb per gram feces. The CRC screening program successfully started, with high participation rates and yield of screening. Now that the program has reached a steady state, there is potential to further optimize the program. Previous studies showed that prior fecal Hb (f-Hb) concentrations just below the FIT cut-off are associated with a higher risk for detection of advanced neoplasia (AN) at subsequent screening rounds. We aim to achieve a better balance between the harms and benefits of CRC screening by offering participants tailored invitation intervals based on prior f-Hb concentrations after negative FIT.

**Methods:**

This mixed-methods study will be performed within the Dutch national CRC screening program and will consist of: (1) a randomized controlled trial (RCT), (2) focus group studies, and (3) decision modelling. The primary outcome is the yield of AN per screened individual in personalized screening vs. uniform screening. Secondary outcomes are perspectives on, acceptability of and adherence to personalized screening, as well as long-term outcomes of personalized vs. uniform screening. The RCT will include 20,000 participants of the Dutch CRC screening program; 10,000 in the intervention and 10,000 in the control arm. The intervention arm will receive a personalized screening interval based on the prior f-Hb concentration (1, 2 or 3 years). The control arm will receive a screening interval according to current practice (2 years). The focus group studies are designed to understand individuals’ perspectives on and acceptability of personalized CRC screening. Results of the RCT will be incorporated into the MISCAN-Colon model to determine long-term benefits, harms, and costs of personalized vs. uniform CRC screening.

**Discussion:**

The aim of this study is to evaluate the yield, feasibility, acceptability and (cost-) effectiveness of personalized CRC screening through tailored invitation intervals based on prior f-Hb concentrations. This knowledge may be of guidance for health policy makers and may provide evidence for implementing personalized CRC screening in The Netherlands and/or other countries using FIT as screening modality.

*Trial registration:* ClinicalTrials.gov, NCT05423886, June 21, 2022, https://clinicaltrials.gov/ct2/show/NCT05423886

**Supplementary Information:**

The online version contains supplementary material available at 10.1186/s12876-023-02670-1.

## Background

In 2014, a national population-based colorectal cancer (CRC) screening program was implemented in the Netherlands. Biennial fecal immunochemical testing (FIT) for hemoglobin is used at a cut-off of 47 µg (µg) hemoglobin (Hb)/g (gram) feces. The CRC screening program successfully started, with high participation rates and yield of screening resulting in a decrease in overall and advanced-stage CRC incidence [1–3]. Now that the program has reached a steady state, there is potential to further optimize the program.

Every year, about 2.2 million people are invited to participate in the Dutch CRC screening program. The participation rate is about 72% [4]. About 4.5% of participants has a positive FIT, meaning they have a fecal hemoglobin (f-Hb) concentration above the pre-set FIT cut-off [4]. Of these participants, about 85% undergo a colonoscopy, with around 40% of these people having a relevant finding (6% CRC and 36% advanced adenoma (AA)) [4]. This implies that about 98% of participants in CRC screening do not experience any benefit from screening; 95.5% of participants because they have a negative FIT and 2.7% because they have a positive FIT without relevant findings at colonoscopy.

Ideally, screening should be offered primarily to those who would benefit most, that is, those who are at high risk of the disease. Personalized screening has been discussed for a long time (about 25 years) [5]. To date, however, such an approach has not taken off, due to the limited predictive power of a number of known risk factors [6]. A risk model that combined genetic information with lifestyle factors, family history and sex had a discriminatory power of 63% for predicting CRC risk [10].

There is increasing evidence that f-Hb concentration is a good predictor of future diagnosis of advanced neoplasia (AN) (Table 1). Models incorporating f-Hb concentrations could reach a discriminatory power of about 80% [6–10]. The major advantage of this predictive factor is that the f-Hb concentration is automatically obtained within FIT-based CRC screening programs and thus is readily available information. The likelihood that the integration of tailored invitation intervals based on prior f-Hb concentration after negative FIT lowers the participation rate is therefore smaller than if another (not automatically obtained) risk factor would be used to personalize CRC screening. Sex and age are also automatically registered, but their predictive value is much lower than the f-Hb concentration (odds ratios for AN: 1.6 (male sex) and 0.9–1.1 (increasing age) vs. 2.5–21.8 (increasing f-Hb concentrations), respectively [7]). In addition, a strong association was observed between the measured f-Hb concentration in participants with a negative FIT and the risk of developing interval CRC in the Dutch CRC screening program [11]. Interval CRC is defined as CRC diagnosed after a negative FIT and before invitation to the next screening round [12]. Participants in the category with an f-Hb concentration just below the FIT cut-off (15–46.9 µg Hb/g feces) are 13 times more likely to develop an interval CRC compared to participants with an unmeasurable f-Hb concentration (0 µg Hb/g feces) [*personal communication*].

**Table 1:** Risk of AN and/or CRC in subsequent screening rounds in high-risk individuals compared to low-risk individuals

Program	FIT cut-off	Comparison high- vs. low-risk individuals	Main outcome	Risk of AN and/or CRC in subsequent round
Dutch pilot studies ^14^	10 µg Hb/g feces	8–10 µg Hb/g feces vs. 0 µg Hb/g feces	AN	Hazard ratio: 8
Flemish CRC screening program^15^	15 µg Hb/g feces	Males aged 74 and 200 µg Hb/g feces vs. females aged 56 and 15 µg Hb/g feces	CRC	Odds ratio: 15
Dutch CRC screening program^16^	47 µg Hb/g feces	15–46.9 µg Hb/g feces vs. 0 µg Hb/g feces	AN	Odds ratio: 23
Scottish CRC screening program^17^	80 µg Hb/g feces	60.0–79.9 µg Hb/g feces vs. 0.0–19.9 µg Hb/g feces	AN	Odds ratio: 38

*CRC* colorectal cancer, *FIT* fecal immunochemical testing, *µg Hb/g* microgram hemoglobin per gram, *AN* advanced neoplasia

Almost half of all interval CRCs occur in a small group of participants (3.5%) with an f-Hb concentration between 15 and 46.9 µg Hb/g feces [13]. Two-thirds of these cancers occur in the second year after screening [13]. This means that one-third of interval CRCs could potentially have been prevented by inviting only 3.5% of participants to screening one year earlier. Based on more recent data, we expect around 85% of participants to have an f-Hb concentration of 0 µg Hb/g feces and thus to be at lowest risk of developing an interval CRC. If the interval between invitations for this group would be extended by one year, this would represent a 40% reduction in the screening burden for the population as a whole.

Now that the FIT-based CRC screening program has been fully rolled out in the Netherlands, has high participation rates and shows favorable results, there is potential for further optimization of the CRC screening program. We designed a mixed-methods study consisting of: (1) a parallel group, two-arm, superiority randomized controlled trial (RCT), (2) focus group studies, and (3) decision modelling. The aim of this mixed-methods study is to identify the yield and (cost-) effectiveness of personalized CRC screening, whether it could be feasible within population-based CRC screening programs, and whether the population is able to understand and accept it.

## Methods/design

### Objectives

The aim of this study is to evaluate the yield, feasibility, acceptability and (cost-) effectiveness of personalized CRC screening through tailored invitation intervals based on prior f-Hb concentrations. Table 2 describes the aims, outcomes, and designated components of the study.

**Table 2:** Aims, outcomes and designated components of the PERFECT-FIT study

Aim	Outcome	Component of the mixed-methods study
Yield	Detection rate	RCT
Effectiveness	Detection of AN	RCT
	Cost-effectiveness	Decision modeling
	Long-term outcomes (incidence & mortality)	Decision modeling
Feasibility	Participation rate	RCT
	Information needs in personalized screening	Focus group I
Acceptability	Information needs in personalized screening	Focus group I
	Perspectives on personalized screening	Focus groups II and III

*RCT* randomized controlled trial, *AN* advanced neoplasia, *PERFECT-FIT* personalized colorectal cancer screening: effectiveness of tailored intervals based on prior f-Hb concentration in a FIT-based colorectal cancer screening program

The primary objective of this study is to compare the yield (detection rate; DR) of AN per participant of personalized CRC screening (intervention arm) to uniform biennial CRC screening (control arm). AN is defined as AA or CRC. AA is defined as an adenoma with high grade dysplasia, and/or > 25% villous component, and/or ≥ 10 mm diameter. The DR is defined as the number of individuals with AN per 1000 screened individuals. Currently, advanced serrated polyps (ASPs) are not yet considered as relevant findings of the Dutch FIT-based screening program. However, this could change in the near future, due to new insights into the relevance of the serrated pathway in carcinogenesis. If ASPs are added to the relevant findings of the national CRC screening program, we will also evaluate the yield of the RCT with an updated definition of AN (AA + ASP + CRC).

The secondary objectives are to determine perspectives on, acceptability of and adherence to personalized CRC screening. Furthermore, we aim to evaluate the (cost-) effectiveness of personalized CRC screening compared to the current screening strategy.

This study was approved by the Health Council and fell under the Population Research Act. It was registered at Clinical Trials (NCT05423886) and started on October 14th, 2022. Additional file 1: Table 1 contains all items from the World Health Organization Trial Registration Data Set.

### Study design

**Fig. 1:**
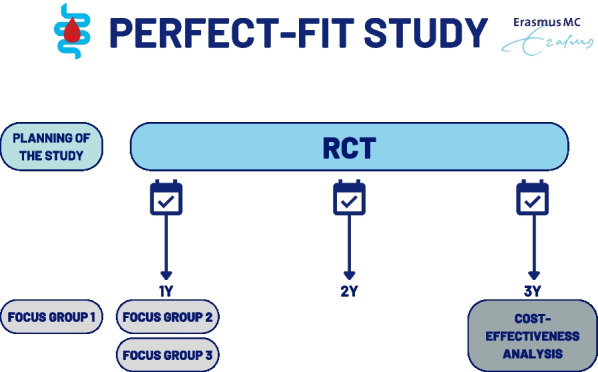
Time schedule of the PERFECT-FIT study. *FIT* fecal immunochemical testing, *RCT* randomized controlled trial, *PERFECT-FIT* personalized colorectal cancer screening: effectiveness of tailored intervals based on prior f-Hb concentration in a FIT-based colorectal cancer screening program

This study is a mixed-methods study consisting of three parts: (1) a parallel group, two-arm, superiority randomized controlled trial (RCT), (2) focus group studies, and (3) decision modelling. This study will be performed over a time period of three years (Fig. 1). A concise time schedule can be found in Additional file 1: Table 2.

## RCT

### Outcomes

We will conduct a prospective, parallel group, two-arm, superiority RCT within the Dutch national, population-based CRC screening program to evaluate the yield of personalized CRC screening by determining the DR of AN (and potentially the updated definition of AN including ASPs) in the intervention and control arm. Furthermore, feasibility will be determined by comparing participation rates between the intervention and control arm.

### Study procedures

The design and logistics of this proposed study will be embedded in the nationwide FIT-based CRC screening program. Screening-eligible individuals with a prior negative FIT (irrespective of screening round) within the Dutch CRC screening program will be included. These individuals will have had a negative FIT ≤ 8 months before inclusion and will have a maximum age of 72, in order for them to undergo at least one more round of screening after participating in the RCT. Individuals will be randomly selected by the CRC screening authority (Bevolkingsonderzoek-Nederland; BVO-NL) from the Mid-West area in the Netherlands.

Individuals who meet the inclusion criteria will be approached by the screening organization (BVO-NL) to participate in the study. Information about the trial will be provided to participants through an information leaflet. Participants will receive the information leaflet by mail, including an informed consent form and a return envelope. General practitioners in the relevant region will receive additional information about the RCT. All individuals will be asked to give informed consent and participate in scientific research, both in the intervention and control group. If individuals choose not to participate, no reminder will be sent and they will receive a standard invitation for screening conform current practice.

**Fig. 2:**
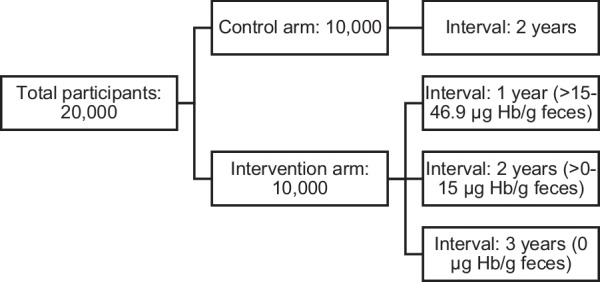
Randomization of participants in the RCT. *µg Hb/g* microgram hemoglobin per gram, *RCT* randomized controlled trial

After providing informed consent, participants will be randomized 1:1 to the control or intervention arm by block randomization according to a computer-generated randomization schedule using permuted blocks. Block sizes will not be disclosed for privacy purposes. Participants will be randomized using R version 4.0.2. Concealment of allocation will be ensured by data transmission through a digital research environment. All participants will be informed whether they have been randomized to the control or intervention arm and will receive a notification letter regarding their invitation interval. Participants in the control arm will receive an invitation to perform FIT at the regular invitation interval, after two years of their prior negative FIT. Individuals in the intervention arm receive information on their prior f-Hb concentration and their corresponding invitation intervals (Fig. 2). They are notified on whether they had little (> 15–46.9 µg Hb/g feces), very little (> 0–15 µg Hb/g feces), or no blood in their stool (0 µg Hb/g feces). They will receive an invitation to perform FIT at the designated invitation interval corresponding with their f-Hb concentration (little blood: 1 year; very little blood: 2 years; no blood: 3 years, Fig. 2).

If an individual does not respond to the invitation, a reminder will be sent after six weeks, conform current practice. Study participants will receive the result of the FIT (negative or positive) according to current practice and in case of a positive FIT also an invitation for an intake appointment for a colonoscopy. The existing IT infrastructure of the CRC screening program, ScreenIT, will be used and adjusted to facilitate allocating personalized invitation intervals within the screening process.

After all participants have performed their FIT within the study, they return to the regular CRC screening program and will again be invited after two years to perform FIT if appropriate.

### Sample size calculation

The power calculation is based on the main endpoint of this study: the yield (DR) of AN (CRC + AA) in the control arm versus the intervention arm. To detect a difference in DR of 0.5% between the intervention and control arm, 20,000 FIT participants are needed. With 20,000 inclusions, we have sufficient power to demonstrate a difference in detection rate of 2.2% in the intervention arm vs. 1.7% in the control arm. Given the high adherence rates of previous participants to subsequent screenings (93%), we conservatively assume that 40% of the invited population is willing to participate in this trial. This means that 50,000 individuals need to be invited to this RCT to demonstrate superiority in yield of risk-based screening. However, if participation rates are lower than expected, more invitations will be sent out until we have reached the total of 20,000 inclusions.

### Data management

All data will be entered electronically by scanning a barcode. The original informed consent forms will be entered into the system and kept on file at the study site. Files are stored in numerical order in a safe, accessible location. Participant records will be retained for at least 15 years after study completion. All reports, data collection, trial and administrative forms will be identified only by an encoded ID number to ensure participant confidentiality. All records with names or other personal identifying information, such as a locator form or informed consent form, are stored separately from study records with ID numbers. All local databases will be protected with password-protected access systems. Forms, spreadsheets, logbooks, and other lists that link participant IDs to other identifying information are stored in a separate locked file in a restricted area. The datasets generated and/or analyzed in this study are not publicly available, but are available on request from BVO-NL. A data transfer agreement will be drawn up in the event of data sharing between BVO-NL and the PERFECT-FIT study team. Data Integrity is enforced through a Data Management Plan; data is owned by BVO-NL and is protected according to the General Data Protection Regulation and other applicable guidelines.

### Study procedures: logistics

1. A study invitation letter will be sent to a selection of screen-eligible individuals who had a negative FIT ≤ 8 months earlier and are still eligible for a subsequent screening round. Invitation letters are sent out in batches of 10,000 invitations. The study invitation will include an information letter and an informed consent form (for the RCT as well as focus groups). Invitees who wish to participate in the study send the informed consent form to the investigators.
2. Informed consent will be returned in a prepaid, pre-addressed return envelope that is sent to the researchers. The barcode on the informed consent will be scanned by one researcher and will be checked by a second researcher.
3. All patients who consent for participation and meet the inclusion criteria will be randomized into either the control or intervention arm by using 1:1 block randomization. No blinding will be performed, as both the investigators and the participants will be informed of the assigned invitation interval. Information on informed consent and randomization of study participants is stored in the eCRF CASTOR.
4. BVO-NL supplies information on f-Hb concentrations of participants that gave consent to participate in the RCT. The researchers assign a screening interval to the participant based on their assigned group and, if applicable, prior f-Hb concentration.
5. Study participants will receive a confirmation letter, stating when the client will be invited again according to the study design (intervention arm: 1, 2 or 3 years and control arm: 2 years).
6. Study participants will receive their FIT within the RCT and will perform the FIT conform the regular screening process.
7. During the study, only the invitation interval of study participants in the intervention arm (1 and 3 years) will be changed. Study participants will receive the regular CRC screening program outcome letter (negative FIT at a cut-off of 47 µg Hb/g feces or positive FIT with an invitation for a follow-up colonoscopy). After participating in the study, all study participants will return to the regular screening program and will be invited to participate in CRC screening two years after the previous invitation date, unless the participant had a positive FIT and was referred for colonoscopy.
8. Individuals returning their consent forms too late (> 3 weeks after receiving their information leaflet and informed consent form) will be excluded from the study and thus follow the regular screening process.
9. A monitoring report provided by BVO-NL will be used to track the progress of the study (including invitations sent and participation rate). If needed, the number of invitations sent will be expanded to reach 20,000 inclusions.
10. At three time points during the study (i.e. 1, 2 and 3 years after inclusion), researchers will receive a report of results from participants who have given informed consent for the study. From study invitees who did not participate in the study (no informed consent), the researchers will receive a report with aggregated/anonymous data (i.e., information on age, sex and f-Hb concentration) to be able to assess generalizability of the results to the entire population.
11. Upon completion of the study, BVO-NL will verify that the study invitees will receive another invitation to the CRC screening program, two years after performing their FIT within the study, according to current practice (unless the participant had a positive FIT).
12. In case participants have logistical questions about the study or the regular CRC screening program, they can visit the study website or ask them by e-mail. There will also be a telephone line available for questions, which will be answered by the researchers of the Erasmus MC.

## Focus group studies

At three time points during the study, a focus group study will be conducted.

### Focus group I

The first focus group study aims to gain insight in information needs among individuals eligible for CRC screening (i.e., acceptability and feasibility of personalized CRC screening). Individuals’ perspectives on personalized CRC screening and information needed to make a well-informed choice whether to participate or not are unknown. The study population consists of individuals that are eligible for CRC screening (i.e. men and women aged 55 to 75 years). This focus group will be conducted online. As this is a qualitative focus group, no formal sample size calculation is required. We aim at including a minimal number of 4 individuals and a maximum of 8 individuals per focus group. Inclusions are continued until thematic saturation is reached; we expect to reach saturation after 3 focus groups (i.e., a minimum number of 12 participants, a maximum number of 24 individuals).

### Focus group II and III

Focus group studies two and three are conducted during the RCT (Fig. 1). In these focus group studies, we would like to determine the acceptability of personalized CRC screening. We deliberately chose not to add an additional questionnaire to assess individuals' view on personalized screening, as this may jeopardize participation. It is important to obtain additional information on individuals' motivations for participating in personalized CRC screening, as well as their perspectives on tailored screening intervals. Focus groups will be conducted in two groups:

- among participants in the intervention arm with a 1-year screening interval;
- among participants in the intervention arm with a 3-year screening interval.

An informed consent form for the focus groups is added to the information leaflet and informed consent form for the RCT. Those individuals that give their consent will be invited for the focus groups when randomized in the intervention arm and having received an invitation interval of 1 or 3 years. Moderators will consist of one of the study coordinators and an independent moderator.

All focus groups will be audio recorded (starting after introduction and verbal consent for recording). The recordings will be transcribed with all identifiers removed. Recordings will be transcribed by an experienced typist as soon as possible after the focus groups. Subsequently, the data will be coded manually and managed using NVivo software. Coding will be translated to English. Analysis will be performed using a framework analysis, a qualitative analytic technique [18].

## Decision modelling

We will use the well-established MIcrosimulation SCreening ANalysis for CRC (MISCAN-Colon) model [19, 20] to estimate harms, benefits, resources and costs of uniform screening with a biennial interval and compare that with those of personalized screening intervals of 1, 2 or 3 years based on prior f-Hb concentrations.

Outcome of the modelling study is the long-term (cost-) effectiveness of personalized screening by using prior f-Hb concentrations. Long-term outcomes include CRC incidence, CRC-related mortality, (quality-adjusted [QA]) life-years [LYs] gained, false-positive tests, colonoscopy complications, and costs, which will be compared for personalized screening versus uniform screening in the Dutch population.

MISCAN-colon was developed by the Department of Public Health of Erasmus MC to evaluate the cost-effectiveness of different CRC screening policies, and it has been used to inform CRC screening policy in the Netherlands, the United States, Canada, and Australia [20–23]. In brief, the MISCAN-Colon model simulates the life histories of a large population of individuals from birth to death and has a natural history component that tracks the progression of underlying colorectal disease in the absence of screening. As each simulated individual ages, there is a chance that one or more adenomas may develop depending on age, sex, race and individual risk. Adenomas can progress from small (1–5 mm) to medium (6–9 mm) to large (≥ 10 mm) size, and some may eventually become malignant. A preclinical cancer (i.e., not detected) has a chance of progressing through different stages and may be detected by symptoms at any stage. With screening, adenomas and preclinical cancers may be detected depending on the sensitivity of the screening test for that lesion and, for endoscopic tests, whether the lesion is within reach of the endoscope.

### Cost-effectiveness analysis

First, we will adjust the MISCAN-Colon model to include f-Hb concentration as a predictive factor for CRC. Next, we will validate model-predicted yield of CRC and AA at different screening intervals to those observed in the results of the RCT. If necessary, the model will be adjusted to improve its predictions. Finally, we will use the model to simulate the 2024 Dutch population and follow this population for a lifetime under two screening strategies: one in which the population is screened every 2 years from age 55 to age 75, and one in which the population is screened in the same age range, but with screening intervals varying between 1 and 3 years based on the f-Hb concentration measured at the prior screening round. Benefits, harms and costs will be compared in a formal incremental cost-effectiveness analysis to determine which of the two strategies is optimal from a cost-effectiveness and health care perspective.

## Discussion

The aim of this study is to evaluate the yield, feasibility, acceptability and (cost-) effectiveness of personalized CRC screening through tailored invitation intervals based on prior f-Hb concentrations. This personalized approach could contribute to a better balance between the harms and benefits of CRC screening on both an individual and population level.

Introducing tailored invitation intervals results in both direct and indirect consequences of personalized CRC screening. Direct consequences are the detection of precancerous lesions or CRC at an earlier stage, as well as reduction of the number of interval CRCs in individuals at higher risk for CRC, by offering specific individuals a shorter invitation interval. In the long-term, this could contribute to a lower burden of CRC-related morbidity and mortality. By inviting participants with an f-Hb concentration just below the cut-off (> 15–46.9 µg Hb/g feces) at a shorter interval, it is expected that, compared to uniform CRC screening, slightly more people will test false positive compared to true positives. Still, the balance of benefits and harms in the high-risk group is expected to be at least as favorable as that of individuals in the low-risk groups. In these low-risk groups, less intensive screening intervals ensures lower burden of screening. There will potentially be an increase in the incidence of interval CRCs in this group because participants will be invited to CRC screening one year later. However, our hypothesis is that the reduction in screening burden clearly outweighs the potential small increase in incidence of interval CRCs. Altogether, it is expected that the balance between harms and benefits of personalized CRC screening will be more favorable compared to uniform CRC screening.

Indirect consequences of implementing personalized CRC screening include ethical and communication challenges [24]. When introducing personalized CRC screening to individuals, there could be confusion between screened individuals living in the same household if they are invited after different time intervals. Another disadvantage could be that those individuals who receive a longer invitation interval will experience stress from the longer waiting time, because of the increased risk of interval CRC. Therefore, providing clear and explicit information on the different invitation intervals based on an individual’s risk is of great importance. The focus group studies will provide invaluable information on perspectives on and acceptability of personalized CRC screening that can be used when personalized CRC screening is potentially introduced at a population level.

It is inevitable that the direct and indirect consequences of personalized CRC screening versus uniform CRC screening will need to be assessed, should personalized screening eventually be implemented at the population level. Possible benefits of a personalized screening approach (i.e., increase in detection of AN, decrease in false-positives, overtreatment, etc.) should be monitored closely, as well as predicted long-term outcomes (i.e., CRC incidence, CRC-related mortality, QALY’s gained, cost-effectiveness). If successful, this study will not only provide evidence for personalized CRC screening, but will also be an important benchmark for quality assurance in future implementation of personalized CRC screening, similar as previous pilot studies preceding the implementation of the Dutch CRC screening program have been for the current uniform program [14, 25–29].

Some limitations or our study should be mentioned. The design of our study is fixed and based on the current test (FIT; FOB-Gold; Sentinel Diagnostics, Milan, Italy), cut-off (47 μg Hb/g feces) and age range (individuals aged 55–75) used in the Dutch CRC screening program. Nevertheless, even if the CRC screening program would be modified in terms of test, cut-off or age range, we expect that the results of our study are still relevant: the effect of the risk factor f-Hb holds for all ages, and the literature shows that it also holds for other cut-offs and FIT brands [7, 11, 13–17, 30–32]. Furthermore, even if the decision should be made to use another test instead of FIT, the study is still informative on the acceptability of risk-based screening in general. Obviously, there will always be organizational and political aspects that need to be considered when planning the real-time implementation of personalized CRC screening [24]. Nevertheless, by embedding this study in the current and ongoing CRC screening program in the Netherlands, it is hoped and expected that (most of) these challenges can be overcome.

We expect there are many future directions in personalized CRC screening; more information will become available on outcomes of multiple screening rounds and on well-known risk factors such as age and sex. Furthermore, in the future other risk factors might also be collected by default within the IT infrastructure, such as lifestyle and genetic (i.e., single nucleotide polymorphisms) factors [24]. If we can implement these risk factors in (advanced) prediction models, the risk prediction for personalized CRC screening can be even further improved, for example through better identification and categorization of the risk groups. If this study demonstrates that personalized CRC screening is successful, such a development would only make risk-based screening more favorable than uniform screening.

In conclusion, the aim of this study is to identify the yield and (cost-) effectiveness of personalized CRC screening, whether it could be feasible within population-based programs, and whether the population is able to understand and accept it. This knowledge may be of guidance for health policy makers and may provide evidence for implementing personalized CRC screening in the Netherlands and/or other countries.

## Supplementary Information

**Additional file 1**. Supplementary tables.

## Data Availability

The informed consent form for participants is written in Dutch and is available upon request from the last author. The datasets generated and/or analyzed during this study are not publicly available but are available on request from BVO-NL. Data from BVO-NL can be requested at Bevolkingsonderzoek Nederland via (wetenschappelijkonderzoek@bevolkingsonderzoeknederland.nl).

## Abbreviations

QALY: Quality-Adjusted Life Years
ASP: Advanced serrated polyp
RCT: Randomized controlled trial
CRC: Colorectal cancer
FIT: Fecal immunochemical testing
AA: Advanced adenoma
F-Hb: Fecal hemoglobin
MISCAN: Microsimulation screening analysis
AN: Advanced neoplasia
DR: Detection rate
BVO-NL: CRC Screening Authority (Bevolkingsonderzoek Nederland, BVO-NL)

## Declarations

### Protocol versions

Issue date: July 16th, 2021.

Protocol Amendment Number: 02.

Author(s): ECHB, ETZ, ILV.

Revision Chronology: July 16th, 2021: original; January 28th, 2022: 1st amendment (Primary reason for amendment: alteration of 3-year intervention group (category changed from 0–2.6 µg Hb/g feces to 0 µg Hb/g feces).

### Committees

See Additional file 1: Table 3.

### Communication of trial results

The study results will be released and will be publicly available to the participants, involved organizations, public health officials and the general medical community.
