# Supplementary material for: Personalized colorectal cancer screening: study protocol of a mixed-methods study on the effectiveness of tailored intervals based on prior f-Hb concentration in a fit-based colorectal cancer screening program (PERFECT-FIT)
Source: BMC Gastroenterol. 2023 Feb 22;23:45. doi: 10.1186/s12876-023-02670-1 (PMC9948315; doi:10.1186/s12876-023-02670-1)
Supplement: Supplementary file 1 — Additional file 1. Supplementary tables. [file 12876_2023_2670_MOESM1_ESM.docx]

**Supplementary materials**

| **Data category** | **Information** |
| --- | --- |
| Primary registry and trial identifying number | ClinicalTrials.gov  NCT05423886 |
| Date of registration in primary registry | June 21, 2022, |
| Source(s) of monetary of material support | Dutch Digestive Foundation (Maag-, lever-, darmstichting, MLDS). Project number: WO 19-44. |
| Primary sponsor | Erasmus University Medical Center (Erasmus MC)  Contact name: Prof. dr. I. Lansdorp-Vogelaar  Address: Dr. Molewaterplein 40, 3015 GD, Rotterdam, The Netherlands  Building: NA-24, room 18 |
| Contact for public queries | Prof. dr. I. Lansdorp-Vogelaar, [i.vogelaar@erasmusmc.nl](mailto:i.vogelaar@erasmusmc.nl)  Dr. E. Toes-Zoutendijk,  [e.toes-zoutendijk@erasmusmc.nl](mailto:e.toes-zoutendijk@erasmusmc.nl)  Drs. L. de Jonge,  [l.dejonge.3@erasmusmc.nl](mailto:l.dejonge.3@erasmusmc.nl) |
| Contact for scientific queries | Prof. dr. I. Lansdorp-Vogelaar, [i.vogelaar@erasmusmc.nl](mailto:i.vogelaar@erasmusmc.nl)  Dr. E. Toes-Zoutendijk,  [e.toes-zoutendijk@erasmusmc.nl](mailto:e.toes-zoutendijk@erasmusmc.nl)  Drs. L. de Jonge,  [l.dejonge.3@erasmusmc.nl](mailto:l.dejonge.3@erasmusmc.nl) |
| Public title | PERFECT-FIT study |
| Scientific title | PERFECT-FIT; personalized colorectal cancer screening: effectiveness of tailored intervals based on prior f-Hb concentration in a FIT-based colorectal cancer screening program |
| Countries of recruitment | The Netherlands |
| Health condition(s) or problem(s) studies | Colorectal cancer (screening) |
| Intervention(s) | Effectiveness of personalized CRC screening vs. uniform CRC screening |
| Key inclusion and exclusion criteria | Screening-eligible individuals with a prior negative FIT (irrespective of screening round) These individuals should have had a negative FIT ≤8 months before inclusion and will have a maximum age of 72, in order for them to undergo at least one more round of screening after participating in the RCT. All other individuals are excluded. |
| Study type | Randomized controlled trial  Intervention model: 1:1 block randomization  Masking: not blinded  Primary purpose: prevention |
| Date of first enrolment | October 14^th^, 2022 |
| Target sample size | 20.000 |
| Recruitment status | Recruiting |
| Primary outcome(s) | Yield (detection rate) of advanced neoplasia per participant of personalized CRC screening (intervention arm) compared to uniform biennial CRC screening (control arm) |
| Key secondary outcomes | Perspectives on, acceptability of and adherence to personalized CRC screening; cost-effectiveness of personalized CRC screening compared to the current screening strategy. |

Supplementary Table 1: items from the World Health Organization Trial Registration Data.

| **Tasks** |  | **Year 1** | | **Year 2** | | **Year 3** | | **Year 4** | | |  |
| --- | --- | --- | --- | --- | --- | --- | --- | --- | --- | --- | --- |
|  | 2021-2 | 2022-1 | 2022-2 | 2023-1 | 2023-2 | 2024-1 | 2024-2 | | 2025-1 | 2025-2 | |
| Permission Population Research Act |  |  |  |  |  |  |  | |  |  | |
| Focus group |  |  |  |  |  |  |  | |  |  | |
| Adjustment IT infrastructure |  |  |  |  |  |  |  | |  |  | |
| Invitation to RCT |  |  |  |  |  |  |  | |  |  | |
| Invitation to FIT,1 year |  |  |  |  |  |  |  | |  |  | |
| Invitation to FIT, 2 year |  |  |  |  |  |  |  | |  |  | |
| Invitation to FIT, 3 year |  |  |  |  |  |  |  | |  |  | |
| Analysis outcomes |  |  |  |  |  |  |  | |  |  | |

Supplementary Table 2: Time schedule of the PERFECT-FIT study.

| **Organizational structure and responsibilities** | **Contact** |
| --- | --- |
| Principal Investigator (PI) and Coordinating Investigators (CI) | PI: Prof. dr. I. Lansdorp-Vogelaar, [i.vogelaar@erasmusmc.nl](mailto:i.vogelaar@erasmusmc.nl)  CI: Dr. E. Toes-Zoutendijk,  [e.toes-zoutendijk@erasmusmc.nl](mailto:e.toes-zoutendijk@erasmusmc.nl)  CI: Drs. L. de Jonge,  [l.dejonge.3@erasmusmc.nl](mailto:l.dejonge.3@erasmusmc.nl) |
| Design and conduct of PERFECT-FIT | E. Toes-Zoutendijk  M. C. W. Spaander  E. Dekker  F. J. van Kemenade  A. J. van Vuuren  C. R. B. Ramakers  I. D. Nagtegaal  M. E. van Leerdam  I. Lansdorp-Vogelaar |
| Preparation of protocol and revisions | I. Lansdorp-Vogelaar  E. Toes-Zoutendijk  E. C. H. Breekveldt |
| Preparation of CRFs [Case Report Forms] | L. de Jonge  E. Toes-Zoutendijk |
| Organizing steering committee meetings | L. de Jonge  E. Toes-Zoutendijk |
| Managing CTO [Clinical Trials Office] | L. de Jonge  E. Toes-Zoutendijk |
| Members of TMC [Trial Management Committee] | L. de Jonge  E. Toes-Zoutendijk |
| Steering committee (SC) | L. de Jonge  E. Toes-Zoutendijk  E. C. H. Breekveldt  M. C. W. Spaander  E. Dekker  F. J. van Kemenade  A. J. van Vuuren  C. R. B. Ramakers  I. D. Nagtegaal  M. E. van Leerdam  I. Lansdorp-Vogelaar |
| Trial Management Committee (TMC) | I. Lansdorp-Vogelaar  E. Toes-Zoutendijk  L. de Jonge |
| Data Manager | P. de Vries, [p.devries.3@erasmusmc.nl](mailto:p.devries.3@erasmusmc.nl) |

Supplementary Table 3: overview of committees involved in the PERFECT-FIT study
